# Supplementary material for: Associations of COVID-19 Knowledge and Risk Perception with the Full Adoption of Preventive Behaviors in Seoul
Source: Int J Environ Res Public Health. 2021 Nov 18;18(22):12102. doi: 10.3390/ijerph182212102 (PMC8623986; doi:10.3390/ijerph182212102)
Supplement: Supplementary file 1 [file ijerph-18-12102-s001.zip › ijerph-1460595-supplementary.pdf]

Table S1. Levels of COVID-19 knowledge among Seoul's citizens (n = 3,000).

	***n* (%)**			
	**All**	Men (n =1463)	Women (n = 1537)	p^*
**Q1. Common symptoms of COVID-19 include fever, coughing, and respiratory difficulty, but in rare cases, nausea and diarrhea can occur.**	**2224 (74.1)**	**1026 (70.1)**	**1198 (77.9)**	**<0.001**
**Q2. The incubation period of COVID-19 is 1–3 days.**	**2056 (68.5)**	**990 (67.7)**	**1066 (69.4)**	**0.320**
**Q3. COVID-19 can be diagnosed by "Holding your breath for 10 seconds." (If you can hold your breath for 10 seconds, you are not infected with COVID-19.)**	**2317 (77.2)**	**1116 (76.3)**	**1201 (78.1)**	**0.225**
Q4. COVID-19 spreads through respiratory droplets produced through coughing and sneezing.	2880 (96.0)	1397 (95.5)	1483 (96.5)	0.163
**Q5. Eating garlic can prevent COVID-19.**	**2298 (76.6)**	**1140 (77.9)**	**1158 (75.3)**	**0.095**
**Q6. COVID-19 spreads through close contact with an infected person.**	**2768 (92.3)**	**1334 (91.2)**	**1434 (93.3)**	**0.030**
**Q7. An ultraviolet sterilizer can be used to prevent COVID-19.**	**1244 (41.5)**	**597 (40.8)**	**647 (42.1)**	**0.474**
Q8. Hand washing and maintaining good personal hygiene can prevent COVID-19.	2847 (94.9)	1367 (93.4)	1480 (96.3)	0.000
Q9. Masks are useful in preventing the spread of respiratory droplets when coughing.	2916 (97.2)	1418 (96.9)	1498 (97.5)	0.372
**Q10. High-temperature dryers can prevent COVID-19.**	**2626 (87.5)**	**1262 (86.3)**	**1364 (88.7)**	**0.040**
Q11. If you experience symptoms after coming into direct contact with someone who may have been infected, you must receive a screening test at a nearby health center.	2845 (94.8)	1380 (94.3)	1465 (95.3)	0.221
Q12. The possibility of COVID-19 progressing into a serious illness is high for the elderly and people with chronic illnesses.	2902 (96.7)	1406 (96.1)	1496 (97.3)	0.058
**Q13. Staying in the sun can prevent COVID-19.**	**2276 (75.9)**	**1127 (77.0)**	**1149 (74.8)**	**0.145**
Q14. Children and young people do not need to implement the COVID-19 prevention guidelines.	2919 (97.3)	1416 (96.8)	1503 (97.8)	0.091
**Q15. Antibiotics must be kept at home as they can treat COVID-19.**	**2431 (81.0)**	**1201 (82.1)**	**1230 (80.0)**	**0.149**
**Q16. People who have encountered those infected by COVID-19 must be quarantined at an appropriate location.**	**2737 (91.2)**	**1342 (91.7)**	**1395 (90.8)**	**0.349**
Q17. Generally, the quarantine period is 14 days.	2838 (94.6)	1375 (94.0)	1463 (95.2)	0.146

Notes: Percentages (%) indicate the rate of correct answers to each question, which refers to the obtained individual scores out of 100. Bold characters indicate the questions for which participants obtained scores lower than the high tertile cut-off score of 94.2 points. * denotes the significance of the results on gender differences obtained from the chi-square tests.

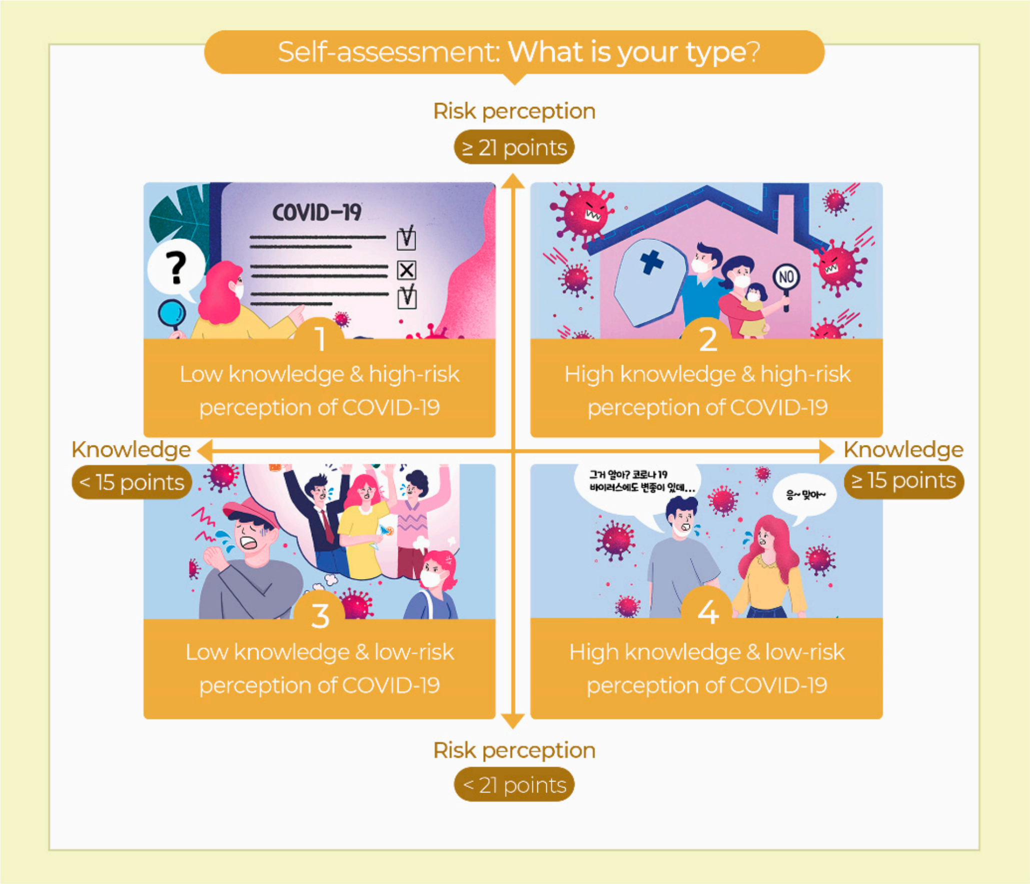

Type 1 -Low knowledge and high-risk perception of COVID-19

You are currently worried about the COVID-19 situation!
Try to ease your anxiety by following the guidelines for mental health.

Type 2 – High knowledge and high-risk perception of COVID-19

You already know a lot about COVID-19!
Please continue to pay attention to any updated information on COVID-19 as you are currently doing.
However, you are also worried about the COVID-19 situation.
Try to ease your anxiety by following the guidelines for mental health.

Type 3 – Low knowledge and low-risk perception of COVID-19

You are a little careless about the current COVID-19 situation!
Please pay more attention to the government guidelines for you and your family.
You still need to focus more on getting COVID-19 information and knowledge; therefore, please recheck some of the survey questions you answered before.

Type 4 – High knowledge and low-risk perception of COVID-19

You already know a lot about COVID-19!
Please continue to pay attention to any updated information on COVID-19 as you are currently doing.
However, you are a little careless about the current COVID-19 situation. Please pay more attention to the government guidelines for you and your family.

Figure S1. Customized messages according to four types based on each total score of COVID-19 knowledge and risk perception.

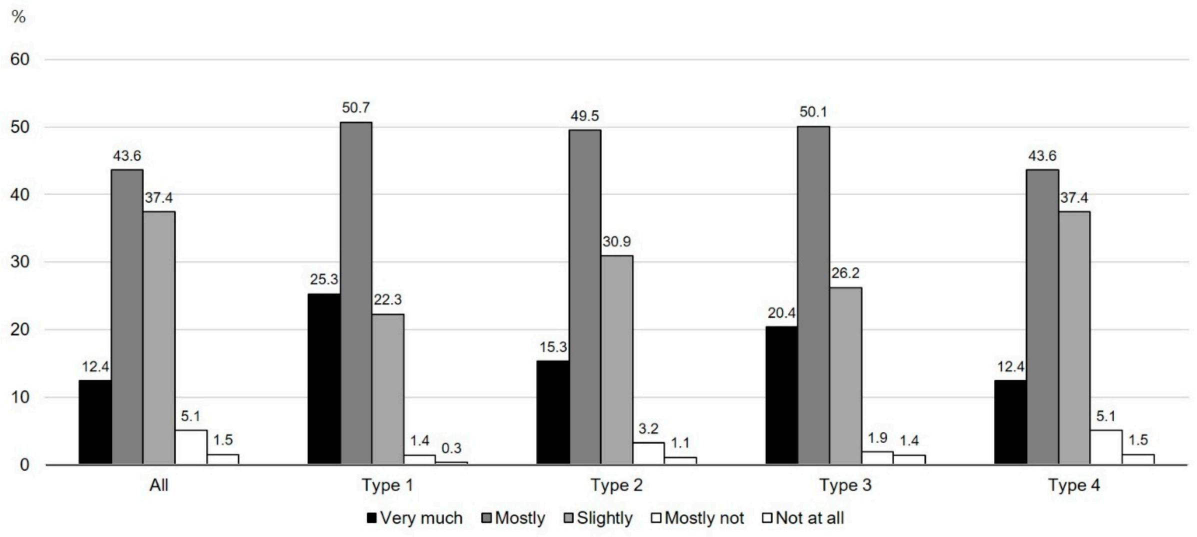

Figure S2. Levels of satisfaction with customized messages by four types among Seoul citizens (***n*** = 3,000).
